# Mr. Davies on the Good Effects of Calomel in a Case of Obstinate Vomiting

**Published:** 1811-04

**Authors:** D. H. Davies

**Affiliations:** 27, Carburton Street, Fitzroy Square


*To the Editors of the Medical and Physical Journal.*

Gentlemen,

You will much oblige me by inserting the following case and observations in your useful miscellany.

——— Payne, a man about the age of fifty, applied to me, some time since, for relief; he stated, that as long as he could remember, he had always been affected with a pain at the pit of the stomach, which was generally worse at one time than another; but that within the last six years of his life he had suffered more violently from it. That about five years since he was attacked with violent vomiting, unattended (as is usual) by a sensation of sickness; he then applied for medical relief, and was recommended to take an emetic occasionally, which he did several times, but without experiencing the least relief, or abatement of vomiting; other medicines were then resorted to by the practitioners who attended him, but without success. Finding himself no better, and the efforts of regular practice unavailing and ineffective, he applied to a *noted* water doctor, who, in addition to his medicines, dicines, strongly recommended the use of stimulants taken into the stomach in the way of food, such as eating a large quantity of mustard, the free use of pepper, &c. this plan of treatment proved, in the end, equally abortive, the vomiting still continued; he frequently brought up the whole of the food that he had recently taken. He says, every act of vomiting is preceded by a great evacuation of wind through the mouth; so much noise has been occasioned by it that it might be heard at a considerable distance, and a very acute pain is always felt after the vomiting. Sometimes a considerable quantity of water is brought up from the stomach, but whether water or food it always appears in a state of violent fermentation. His own description of what is evacuated is, that it is similar to dough in a state of effervescence; his bowels are always in a state of constipation, he frequently has not an evacuation from them oftener than once in six or seven days, and he has frequently been eight days without one. His complaint is strongly marked by dyspeptic symptoms, loss of appetite, dejection of spirits, languor and emaciation. At my first interview with the patient, I was disposed to think his complaint arose from a chronic inflammation of the stomach, but the pain not being aggravated by pressure, nor increased immediately after taking in aliment, I was induced to abandon the idea. It appeared more rational to suppose there must be some obstructing cause preventing the free passage of bile into the intestines, and that in consequence of that obstruction, part of the bile might regurgitate into the stomach, the coats of which being irritable, would endeavour to get rid of the offending cause by vomiting, and that, with the bile, the other contents of the stomach would naturally be thrown off. Under this impression, as emetics had proved inefficacious, I directed gr. iss. of calomel to be taken night and morning, and the use of friction at the pit of the stomach, wishing, by the exhibition of mercury, to induce a different action in the system, and, if possible, to remove the morbid cause of the disease. He continued this plan for four days; on the fifth I saw him, he had had no return of the vomiting, had one alvine evacuation, and his gums were beginning to be slightly sore. I now directed him to take a tonic medicine two or three times a day, with a view to strengthen the tone of his stomach, and desired him to continue the calomel every night at bed time, for a week longer, when I requested to see him again, as he lived at a considerable distance from my residence. At the time that I saw him again, he informed me that in the evening of the day I saw him, he had a little return of the vomiting, but none since, which comprised in the whole since

I first

I first saw him, the space of ten or eleven days, in the course of which time he experienced but one act of vomiting, whereas he had previously been accustomed to feel a return of it about every other day; he had had for the last week a motion every second day, which he had not had for several years before; he has little or no evacuation of wind, his appetite and general health are better; the mercury has made his gums unpleasantly sore; I ordered him to continue the calomel every night as before for a week longer, and to take the tonic medicine. At the time I saw him again, he informed me he had had no return of vomiting, he continued regular in his bowels as before, and had improved in his health. His mouth was much sorer than when I saw him last, I desired him to continue in the same plan for a week; when I saw him again his mouth was extremely sore, he spit a great deal, *no return* of sickness, bowels regularly open, and his appetite better, ordered him to discontinue the mercury, and to take the tonic medicine for a week or ten days. His general appearance and health gradually became more evident, and he is now considerably better than he has been for years; the vomiting appears to be entirely overcome. I have thus simply related the history of a case, which I think rather *singular*, and in which mercury seems to have been of eminent *service;* I am not theorist enough to attempt to explain its mode of action on the system, but its utility in this case was conspicuous. I remain, Gentlemen,

Yours, &c.

D. H. DAVIES.

*27, Carburton Street,*

*Fitzroy Square.*

